# Perioperative outcomes in an age-adapted analysis of the German StuDoQ|Pancreas registry for PDAC

**DOI:** 10.1186/s12893-024-02647-1

**Published:** 2025-01-04

**Authors:** Tengis Tschaidse, Felix O. Hofmann, Bernhard Renz, Maximilian Hungbauer, Carsten Klinger, Heinz J. Buhr, Waldemar Uhl, Sören Torge Mees, Tobias Keck, Christoph Reissfelder, Michael Ghadimi, Jan G. D’Haese, Jens Werner, Matthias Ilmer

**Affiliations:** 1 Department of General, Visceral and Transplantation Surgery, LMU University Hospital Munich, LMU Munich, Munich, Germany; 2 German Cancer Consortium (DKTK), Partner Site Munich; and German Cancer Research Center (DKFZ), Heidelberg, Germany; 3 German Society of General and Visceral Surgery (DGAV), Berlin, Germany; 4 Department of General and Visceral Surgery, St. Josef-Hospital Bochum, Ruhr-University Bochum, Bochum, Germany; 5 Department of General and Visceral Surgery, Dresden-Friedrichstadt General Hospital, Teaching Hospital of the Technical University Dresden, Dresden, Germany; 6 Department of Surgery, University Medical Center Schleswig-Holstein, Campus Lübeck, Lübeck, Germany; 7 Department of Surgery, Universitätsmedizin Mannheim, Medical Faculty Mannheim, Heidelberg University, Mannheim, Germany; 8 Department of General, Visceral, and Paediatric Surgery, University Medical Center, Göttingen, Germany; 9 Department for General, Visceral, Endocrine and Vascular Surgery, Krankenhaus Agatharied GmbH, Hausham, Germany; 10 Department of General, Visceral, and Transplantation Surgery, Hospital of the University of Munich, Ludwig-Maximilians-University (LMU) Munich (Germany), Marchioninistr, 15, München, 81377 Germany

**Keywords:** Age, Early-onset, PDAC, StuDoQ, Early-onset cancer, Pancreatic cancer

## Abstract

**Background:**

Pancreatic ductal adenocarcinoma (PDAC) typically occurs in an older patient population. Yet, early-onset pancreatic cancer (EOPC) has one of the fastest growing incidence rates. This study investigated the influence of age and tumor location on postoperative morbidity and mortality in a large, real-world dataset.

**Methods:**

Patients with confirmed PDAC undergoing pancreatic surgery between 01/01/2014 and 31/12/2019 were identified from the German StuDoQ|Pancreas registry. After categorization into early- (EOPC; < 50 years), middle- (MOPC; 50 -70 years), and late-onset (LOPC; > 70 years), and stratification into pancreaticoduodenectomy (PD) or distal pancreatectomy (DP), differences in morbidity and mortality as well as clinicopathologic parameters were analyzed.

**Results:**

In total, 3011 patients were identified. No difference in the occurrence of postoperative pancreatic fistula (POPF), postpancreatectomy hemorrhage (PPH) or delayed gastric emptying (DGE) between different age groups and resection techniques was detected. However, in patients undergoing PD, major complications (Clavien-Dindo ≥ 3a) were observed more frequently in LOPC (30,7%) than in MOPC (26,2%) and EOPC (16,9%; *p <* 0,01). Mortality almost tripled from EOPC (2,4%) to MOPC (3,6%) to LOPC (6,6%, *p <* 0,01) and significantly higher failure to rescue (FTR) rates could be observed (EOPC 14,3%, MOPC 13,6%; LOPC 21,6%; *p <* 0,05). In centers with DGAV certification for pancreatic surgery, the risk of complications was significantly decreased in PD (OR 0,79; 95% CI 0,65–0,94; *p* = 0,010).

**Conclusion:**

Age has a pronounced impact on the perioperative outcomes after pancreatic resections of PDAC. This effect is more prevalent in PD compared to DP. Pancreatic surgery-specific complications, such as POPF, DGE or PPH do not occur more frequently in the elderly. Overall, the risk of major complications and mortality increases in elderly patients mainly secondary to higher FTR rates.

**Supplementary Information:**

The online version contains supplementary material available at 10.1186/s12893-024-02647-1.

## Introduction

Pancreatic ductal adenocarcinoma (PDAC) is currently the third leading cause of cancer-related death in the USA and is projected to become the second by 2030 [1]. On average, diagnosis of PDAC occurs after the sixth decade of life, but around 5%—10% of patients are diagnosed with 50 years of age or younger [2]. This group can be referred to as early-onset pancreatic cancer (EOPC). While the incidence of early-onset cancer in general seems to be increasing, EOPC has one of the fastest growing incidence rates observed [3]. Even though EOPC aggregates only 1%—5% of the total deaths from pancreatic cancer, it accounts for 20% to 30% of the total numbers of years-of-life-lost caused by the disease [2]. Thus, understanding the perioperative outcomes and risk factors specific to EOPC is imperative to improve overall management and patient outcomes. Due to this limited knowledge about EOPC, it is questionable if established risk factors, derived mostly from an elder population, are equally applicable for this rare subgroup. In general, patients with EOPC seem to present in more advanced stages with frequent metastases and poor survival [4, 5]. However, a recent analysis in two high volume institutions has demonstrated that in instances when resection in EOPC is feasible, satisfactory oncologic outcomes are achievable, even though patients frequently present with advanced tumors [6]. Still, EOPC remains poorly characterized, necessitating further investigation to optimize treatment strategies for this specific patient subset.

Conversely, by the end of this decade patients aged 65 and older will make up 70% of all patients diagnosed with cancer [7]. For elderly patients with PDAC, available data suggest that with increased age, perioperative risks increase, too [8, 9]. Yet, surgical resection remains the only option for cure in patients with PDAC. Pylorus-preserving pancreaticoduodenectomy (PPPD) or Whipple procedure is necessary to remove PDAC of the head, which accounts for 60–70% of all PDAC. In contrast, distal pancreatectomy (DP) is required for PDAC in the pancreatic body or tail region, which accounts for 20–25% of all PDAC [10].

As perioperative morbidity and mortality differs between PPPD/Whipple and DP, the aim of this study was to compare perioperative outcomes according to resection type while adjusting for different age groups.

## Methods

### StuDoQ|Pancreas registry

Data obtained from the StuDoQ|Pancreas registry, which is maintained by the German Society for General and Visceral Surgery (Deutsche Gesellschaft für Allgemein- und Viszeralchirurgie; DGAV), was retrospectively analyzed. StuDoQ|Pancreas is a registry specifically designed for pancreatic surgery in Germany and was established in September 2013 to assess quality. Over 60 institutions contributed to the registry at the time of this study. Pseudonymized data from participating centers were entered into a web-based tool with automatic plausibility checks. Annual certification involved validation through cross-checking with institutional medical data. Centers can also apply for an additional certification for pancreatic surgery of the DGAV which requires a minimum of 40 pancreatic resections per year [11].

### Study cohort

**Fig. 1:**
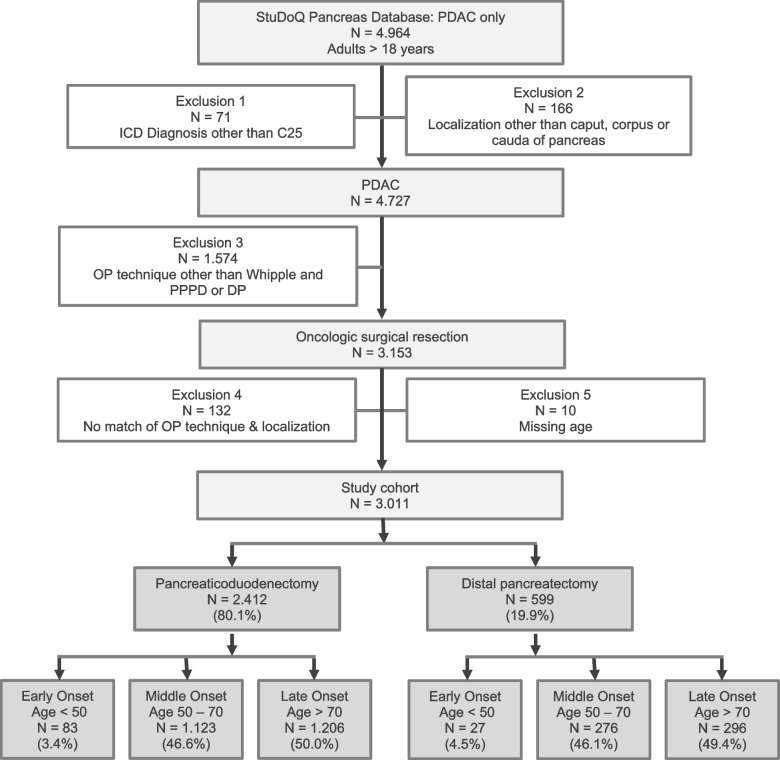
Study profile. ICD = International Classification of Diseases

Eligibility was assessed for all patients who had provided written informed consent at the respective study site and underwent elective surgery between January 2014 and December 2019. All patients with histopathologic diagnosis of PDAC in the head, body or tail who received oncologic resection (Whipple procedure, PPPD or DP) were included. Patients were excluded when localization of PDAC did not match with operational technique. Patients were grouped into Early- (< 50 years) (EOPC), Middle- (50 – 70 years) (MOPC) and Late- (> 70 years) onset (LOPC), and all analyses were conducted after stratification of Whipple operation and PPPD into PD in contrast to DP. The flow chart for the final study cohort can be seen under Fig. 1.

### Data

Apart from surgical procedure, obtained data included baseline parameters (e.g., age, body mass index (BMI), ASA physical status classification system (ASA), preoperative diabetes mellitus (DM), preoperative serum markers (bilirubin, CA19-9, CEA)), perioperative parameters (e.g., length of surgery, postoperative days in hospital and on intensive care unit (ICU), TNM classification (8th edition) and DGAV certification of the surgical department) as well as postoperative outcomes and 30-day complications (e.g., occurrence of postoperative pancreatic fistula (POPF) [12], delayed gastric emptying (DGE) [13], postpancreatectomy hemorrhage (PPH) [14] as defined by the International Study Group of Pancreatic Surgery (ISGPS). Postoperative complications were graded according to the Clavien-Dindo classification [15] and dichotomized into minor (grade ≤ 2) or major (grade ≥ 3a). Failure to rescue (FTR) rates were calculated by dividing deaths in patients by patients with major complications, in accordance with previously described literature [16, 17]. Data extraction and analysis received approval from the StuDoQ steering committee and was conducted in compliance with StuDoQ data protection guidelines (StuDoQ-2019–0010). The Society for Technology, Methods, and Infrastructure for Networked Medical Research (TMF) approved the concept of informed consent and data safety [18]. All data are property of the German Society of General and Visceral Surgery, but can be consulted upon request.

### Statistics

Variables were tested for normality by analyzing histograms and Shapiro–Wilk test. Non-normal distributions were reported in median and compared using Kruskal–Wallis tests. For post hoc testing, Jonckheere-Terpstra test was carried out. Categorial variables were compared using Pearson’s chi square tests or Fisher’s exact tests. Missing values were not included into testing.

Univariable logistic regression was used to investigate the association between potential risk factors and the occurrence of major complications (Clavien-Dindo ≥ 3a) and FTR. Significance level was set at *p <* 0,05 and independent variables which showed a significant association with the occurrence of major complications were then further investigated in a multivariable logistic regression model. Independent variables were entered into the model in forced entry method. Only pre- and intraoperatively collected data were analyzed as independent variables so that the results might be considered for immediate postoperative decision making.

For the statistical analysis, SPSS version 28.0.1.1 (14) was utilized.

## Results

### Patient characteristics

The study cohort included 3011 patients with confirmed PDAC who underwent resection between 2014 and 2019. Of these, 2412 (80,1%) underwent Whipple procedure or PPPD, which was combined to a PD group. In contrast, 599 patients (19,9%) underwent DP. Patients were grouped according to their age in early-onset pancreatic cancer (EOPC; 18–49 years, *n* = 110 (3,7%) in total), middle-onset pancreatic cancer (MOPC, 50–70 years, *n* = 1399 (46,5%) in total) and late-onset pancreatic cancer (LOPC; > 70 years, *n* = 1502 (49,8%) in total).

In the PD group, EOPC consisted of 83 (3,4%), MOPC of 1123 (46,6%) and LOPC of 1206 (50,0%) patients. The DP group comprised 27 patients (4,5%) with EOPC, 276 (46,1%) with MOPC, and 296 (49,4%) with LOPC respectively. ASA scores differed in the PD (*p <* 0,05) and DP (*p <* 0,001) group across the different age groups. In the PD group, patients of the MOPC group tended to have a slightly elevated BMI (*p <* 0,05), whereas in the DP group BMI did not vary. In the PD group, with increasing age, DM was found with a significantly higher incidence preoperatively (*p <* 0,001); this was not the case for patients undergoing DP.

Preoperative bilirubin levels differed in the PD and DP group across the different ages. Tumor markers CEA and CA19-9 tended to be more elevated with increasing age, this difference was only significant for CA19-9 in the PD group. In the PD group, an enlarged pancreatic duct (PD) width was found with increasing age, while the opposite trend was seen in the DP group. There was no difference observed in pancreatic texture. Likewise, neither tumor size nor lymph node status diverged between age groups for both resection types. A summary of all the baseline characteristics can be seen under Table 1.

**Table 1:** Clinicopathologic variables of patients with early-, middle- or late-onset pancreatic adenocarcinomas reported by the StuDoQ database (2014–2019)

**Study cohort**	Total (*n* = 3011)		EOPC (*n* = 110)		MOPC (*n* = 1399)		LOPC (*n* = 1502)		
**Type of surgery**	PD 2412 (80.1%)	DP 599 (19.9%)	Pancreaticoduodenectomy			Distal Pancreatectomy			*P* value
**Onset** **n**			EOPC 83	MOPC 1123	LOPC 1206	EOPC 27	MOPC 276	LOPC 296	
**Age, median** (years)	70.5 [62—77]	70.0 [61—77]	46.0 [43—48]	62.0 [57—67]	77.0 [74—80]	46.0 [45—48]	62.0 [57—67]	77.0 [74—80]	
**ASA**									**P**_**PD**_** < 0.05** **P**_**DP**_**< 0.001**
1	92 (3.8%)	29 (4.8%)	8 (9.6%)	58 (5.2%)	26 (2.2%)	3 (11.1%)	23 (8.3%)	3 (1.0%)	
2	1018 (42.2%)	272 (45.4%)	44 (53.1%)	532 (47.4%)	442 (36.7%)	15 (55.6%)	135 (48.9%)	122 (41.2%)	
3	1253 (51.9%)	287 (47.9%)	31 (37.3%)	522 (46.5%)	700 (58.0%)	9 (33.3%)	115 (41.7%)	163 (55.1%)	
4	48 (2.0%)	11 (1.8%)	-	11 (1.0%)	37 (3.1%)	-	3 (1.1%)	8 (2.7%)	
5	1 (0.01%)		-	-	1 (0.1%)	-	-	-	
Unknown			-	-	-	-	-	-	
**BMI. median** (kg/m^2^)	24.8 [22.6—27.7]	25.1 [22.6—27.9]	24.9 [21.7—28.1]	25.1 [22.8—28.1]	24.7 [22.4—27.2]	25.3 [20.8—28.7]	25.3 [22.6—28.4]	24.9 [22.6—27.5]	**P**_**PD**_** < 0.05** P_DP _= 0.26
**Tumor** **Size** (cm)									P_PD_ = 0.84 P_D__P_ = 0.77
< 2.0	190 (7.9%)	64 (10.7%)	8 (9.6%)	91 (8.1%)	91 (7.5%)	4 (14.8%)	33 (12.0%)	27 (9.1%)	
2.0 – 4.0	884 (36.7%)	185 (30.9%)	26 (31.3%)	413 (36.8%)	445 (36.9%)	7 (25.9%)	84 (30.4%)	94 (31.8%)	
> 4.0	1337 (55.4%)	347 (57.9%)	49 (59.0%)	619 (55.1%)	669 (55.5%)	16 (59.3%)	159 (57.6%)	172 (58.1%)	
Unknown	1 (0.01%)	3 (0.5%)	-	-	1 (0.1%)	-	-	3 (1.0%)	
**Grading**									P_PD_ = 0.18 **P**_**DP**_ **=** **0.05**
G1	87 (3.6%)	27 (4.5%)	5 (6.0%)	41 (3.7%)	41 (3.4%)	4 (14.8%)	16 (5.8%)	7 (2.4%)	
G2	1307 (54.2%)	324 (54.1%)	38 (45.8%)	627 (55.8%)	642 (53.2%)	9 (33.3%)	142 (51.4%)	173 (58.4%)	
G3	935 (38.8%)	208 (34.7%)	34 (41.0%)	411 (36.6%)	490 (40.6%)	11 (40.7%)	95 (34.4%)	102 (34.5%)	
G4	16 (0.7%)	3 (0.5%)	-	10 (0.9%)	6 (0.5%)	1 (3.7%)	1 (0.4%)	1 (0.3%)	
Unknown	67 (2.8%)	37 (6.2%)	6 (7.2%)	34 (3.0%)	27 (2.3%)	2 (7.4%)	22 (8.0%)	13 (4.4%)	
**Lymph node status**									P_PD_ = 0.10 P_DP_ = 0.11
Negative	726 (30.1%)	240 (40.1%)	28 (33.7%)	314 (28.0%)	384 (31.9%)	16 (59.3%)	106 (38.4%)	118 (39.9%)	
Positive	1685 (69.9%)	358 (59.8%)	55 (66.3%)	809 (72.0%)	821 (68.0%)	11 (40.7%)	170 (61.6%)	177 (59.8%)	
Unknown	1 (0.01%)	1 (0.2%)	-	-	1 (0.1%)	-	-	1 (0.3%)	
**Diabetes mellitus**									**P**_**PD**_** < 0.001** P_DP_ = 0.20
No	1710 (70.9%)	444 (74.1%)	37 (88.0%)	818 (72.8%)	819 (67.9%)	22 (81.5%)	294 (73.9%)	218 (73.6%)	
Yes, NIDDM2	327 (13.6%)	84 (14.0%)	2 (2.4%)	127 (11.3%)	198 (16.4%)	3 (11.1%)	32 (11.6%)	49 (16.6%)	
Yes, IDDM2	375 (15.5%)	71 (11.9%)	8 (9.6%)	178 (15.9%)	189 (15.7%)	2 (7.4%)	40 (14.5%)	29 (9.8%)	
Unknown	-	-	-	-	-	-	-	-	
**Preop Bilirubin, median (mg/dl)**	1.9 [0.6—8.5]	0.5 [0.3 -0.7]	1.6 [0.6—7.3]	1.6 [0.5—8.7]	2.2 [0.7—8.5]	0.5 [0.2—0.6]	0.5 [0.3—0.6]	0.5 [0.4—0.7]	**P**_**PD**_** < 0.05** **P**_**DP**_**< 0.05**
Unknown	176 (7.3%)	47 (7.8%)	5 (6.0%)	85 (7.6%)	86 (7.1%)	1 (3.7%)	26 (9.4%)	20 (6.8%)	
**Preop Tumor marker**									
CEA, Median (ng/ml)	3.1 [1.9—5.3]	3.0 [1.8—5.4]	2.8 [1.4—4.0]	3.1 [1.9 -5.2]	3.1 [2.0 -5.4]	2.0 [1.7 -4.2]	2.9 [1.9 -4.9]	3.1 [1.8—6.8]	P_PD_ = 0.14 P_DP_ = 0.19
Unknown	638 (26.5%)	164 (27.4%)	19 (22.9%)	292 (26.0%)	327 (27.1%)	7 (25.9%)	74 (26.8%)	83 (28.0%)	
CA19-9, median (U/ml)	133.0 [32.2—532.8]	76.0 [20.4—412.0]	86.0 [28.4—214.8]	115.1 [28.6—494.7]	158.5 [37.8—649.3]	48.1 [12.9—646.0]	67.1 [17.2—354.0]	92.5 [27.1—488.0]	**P**_**PD**_** < 0.001** P_DP_ = 0.18
Unknown	410 (17.0%)	120 (20.0%)	13 (15.7%)	173 (15.4%)	224 (18.6%)	4 (14.8%)	60 (21.3%)	56 (18.9%)	
**Pancreatic duct width**									**P**_**PD**_** < 0.001** **P**_**DP**_***<* 0.05**
Normal (< 3mm)	882 (26.6%)	251 (41.9%)	32 (38.6%)	442 (39.4%)	408 (33.8%)	8 (29.6%)	111 (40.2%)	132 (44.6%)	
Enlarged (> 3mm)	862 (35.7%)	38 (6.3%)	21 (25.3%)	362 (32.2%)	479 (39.7%)	19 (70.4%)	10 (3.6%)	28 (9.5%)	
Unknown	668 (27.7%)	310 (51.8%)	30 (36.1%)	319 (28.4%)	319 (26.5%)	-	155 (56.2%)	136 (45.9%)	
**Pancreatic texture**									P_PD_ = 0.50 P_DP_ = 0.81
Soft	885 (36.7%)	276 (46.1%)	23 (27.7%)	411 (36.6%)	451 (37.4%)	11 (40.7%)	127 (46.0%)	138 (46.6%)	
Hard	985 (40.8%)	113 (18.9%)	32 (38.6%)	473 (42.1%)	480 (39.8%)	3 (11.1%)	54 (19.6%)	56 (18.9%)	
Unknown	542 (22.5%)	210 (35.1%)	28 (33.7%)	239 (21.3%)	275 (22.8%)	13 (48.1%)	95 (34.4%)	102 (34.5%)	

Baseline characteristics according to PD and DP and according to age group, stratified regarding type of resection (PD vs. DP). Tumor stage and grading derived from postoperative histopathology, pancreatic texture and duct width as evaluated by the surgeon intraoperatively. Continuous data are shown as median, categorial data are shown as absolute (relative). Kruskal—Wallis test, Pearson’s chi square test and Fisher’s exact test used, comparing PD and DP resection within Early-, Middle- and Late-Onset group

*PD* Pancreaticoduodenectomy, *DP* Distal pancreatectomy, *BMI* Body mass index, *EOPC* early-onset pancreatic cancer, *MOPC* middle-onset pancreatic cancer, *LOPC* late-onset pancreatic cancer, *CEA* Tumor marker carcinoembryonic antigen, *CA19-9* Tumor marker carbohydrate antigen 19–9, [] interquartile range

### Perioperative outcomes

The majority of all operations was carried out as open operations (2838/3011; 94%). Only 54 (1,8%) of the total 3011 operations were performed laparoscopically while the rest was either laparoscopically assisted (45/3011; 1,5%) or secondarily converted to open surgery (73/3011, 2,4%); thus, comparisons between open and laparoscopic surgery was not feasible due to the small case numbers. For both PD and DP, the operating time was significantly shorter in EOPC compared to MOPC and to LOPC (*p <* 0,01 and *p <* 0,01; Table 2). In contrast, for both PD and DP, length of hospital stay was longer for patients with LOPC compared to MOPC and to EOPC (*p <* 0,01 and *p <* 0,05; Table 2). For patients receiving PD, length of stay on the ICU got significantly longer from EOPC to LOPC (*p <* 0,01) but did not differ for patients in the DP group (*p* = 0,07; Table 2). Resection (R) status did not differ and comparable rates of R0 resections were achieved and can be seen in Table 2.

**Table 2:** Perioperative variables and outcomes of patients with early-, middle- or late-onset pancreatic adenocarcinoma stratified regarding type of resection (PD vs. DP)

**Type of surgery**	PD (*n* = 2.412)	DP (*n* = 599)	Pancreaticoduodenectomy			Distal Pancreatectomy			*P value*
**Onset** n			EOPC 83	MOPC 1123	LOPC 1.206	EOPC 27	MOPC 276	LOPC 296	
**Median duration** (min)	330 [272—403]	212 [163—269]	352 [290—441]	342 [278—415]	320 [265—384]	240 [165—315]	218.5 [172—277]	203 [155—253]	**P**_**PD**_** < 0.01** **P**_**DP**_**< 0.01**
**Median stay hospital** (days)	16 [12–23]	14.5 [11–22]	15 [12–22]	15 [12–21]	17 [13–24]	12 [11–18]	14 [11–21]	15 [12–23]	**P**_**PD**_** < 0.01** **P**_**DP**_**< 0.05**
**Median stay ICU** (days)	3 [1–5]	1 [1–3]	2 [1–4]	2 [1–4]	3 [1–5]	1 [0 – 2]	1 [1–3]	2 [1–4]	**P**_**PD**_** < 0.01** P_DP_ = 0.07
**Resection status**									P_PD_ = 0.87 P_DP_ = 0.69
R0	1832 (76.0%)	448 (74.8%)	62 (74.4%)	855 (76.1%)	915 (75.9%)	22 (81.5%)	206 (74.6%)	220 (74.3%)	
R1	526 (21.8%)	132 (22.0%)	20 (24.1%)	246 (21.9%)	260 (21.6%)	5 (18.5%)	60 (21.7%)	67 (22.6%)	
R2	26 (1.1%)	5 (0.8%)	1 (1.2%)	11 (1.0%)	14 (1.2%)	-	2 (0.7%)	3 (1.0%)	
Unknown	28 (1.2%)	14 (2.3%)	-	11 (1.0%)	17 (1.4%)	-	8 (2.9%)	6 (2.0%)	
**CR-POPF**									P_PD_ = 0.82 P_DP_ = 0.20
No	2221 (92.1%)	458 (76.5%)	78 (94.0%)	1033 (92.0%)	1110 (92.0%)	24 (88.9%)	205 (74.3%)	229 (77.4%)	
Yes	191 (7.9%)	141 (23.5%)	5 (6.0%)	90 (8.0%)	96 (8.0%)	3 (11.1%)	71 (25.7%)	67 (22.6%)	
**CR-PPH**									P_PD_ = 0.82 P_DP_ = 0.97
No	2203 (91.3%)	578 (96.5%)	77 (92.8%)	1022 (91.0%)	1104 (91.5%)	26 (96.3%)	266 (96.4%)	286 (96.6%)	
Yes	209 (8.7%)	21 (3.5%)	6 (7.2%)	101 (9.0%)	102 (8.5%)	1 (3.7%)	10 (3.6%)	10 (3.4%)	
**CR-DGE**									P_PD_ = 0.14 P_DP_ = 0.06
No	2172 (90.0%)	572 (95.5%)	80 (96.4%)	1011 (90.0%)	1081 (89.6%)	27 (100%)	268 (97.1%)	277 (93.6%)	
Yes	240 (10.0%)	27 (4.5%)	3 (3.6%)	112 (10.0%)	125 (10.4%)	-	8 (2.9%)	19 (6.4%)	
**Complication**									**P**_**PD**_** < 0.01** P_DP_ = 0.18
None/Minor (Clavien-Dindo < 3a)	1734 (71.9%)	459 (76.6%)	69 (83.1%)	829 (73.8%)	836 (69.3%)	24 (88.9%)	215 (77.9%)	220 (74.3%)	
Major (Clavien-Dindo ≥ 3a)	678 (28.1%)	140 (23.4%)	14 (16.9%)	294 (26.2%)	370 (30.7%)	3 (6.3%)	61 (22.1%)	76 (25.7%)	
**Mortality**									**P**_**PD**_** < 0.01** P_DP_ = 0.13
No	2290 (94.9%)	588 (98.2%)	81 (97.6%)	1083 (96.4%)	1126 (93.4%)	27 (100%)	274 (99.3%)	287 (97.0%)	
Yes	122 (5.1%)	11 (1.8%)	2 (2.4%)	40 (3.6%)	80 (6.6%)	-	2 (0.7%)	9 (3.0%)	
**FTR**									**P**_**PD**_** < 0.05** P_DP_ = 0.21
	122 (18.0%)	11 (7.9%)	2 (14.3%)	40 (13.6%)	80 (21.6%)	-	2 (3.3%)	9 (11.8%)	

Overall outcomes according to PD and DP and according to age group. Continuous data are shown as median, categorial data are shown as absolute (relative). Kruskal- Wallis test, Pearson’s chi square test and Fisher’s exact test used, comparing PD and DP resection within Early-, Middle- and Late-Onset group. Post hoc testing was calculated with Jonckheere-Terpstra test

*ICU* intensive care unit, *POPF* postoperative pancreatic fistula, *PPH* postpancreatectomy hemorrhage, *DGE* delayed gastric emptying

**Fig. 2:**
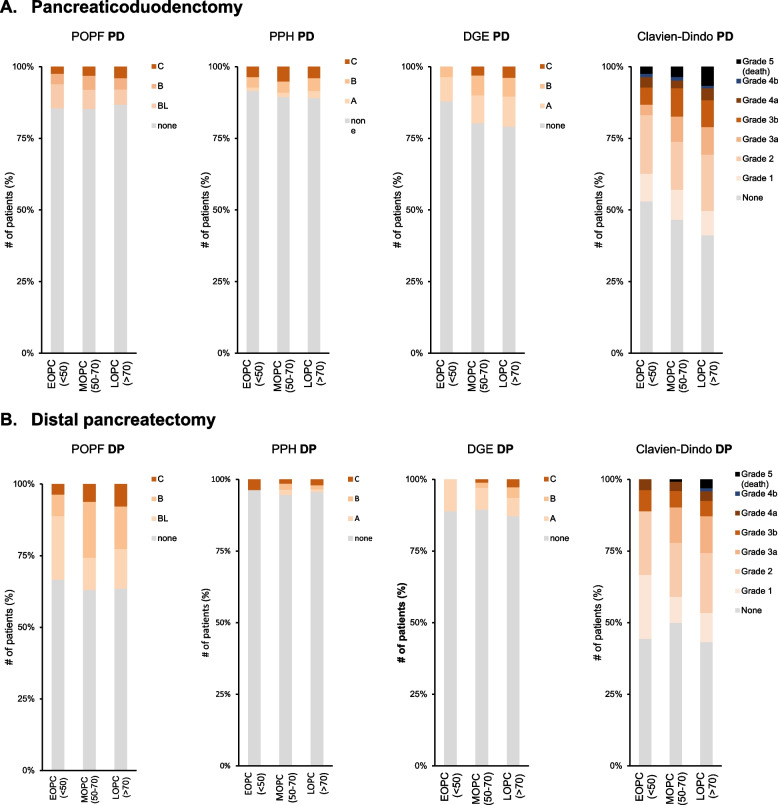
Rates of complications. Rate and grade of complications following PD (**A**) and DP (**B**) adjusted for age. *POPF* = postoperative pancreatic fistula, *PPH* = postpancreatectomy hemorrhage, *DGE* = delayed gastric emptying

We did not detect a difference in the occurrence of POPF, PPH or DGE between different age groups and resection techniques (Table 2, Fig. 2). However, in patients undergoing PD, overall complications graded after Clavien-Dindo were observed more frequently in LOPC compared to MOPC and EOPC (*p <* 0,01; Table 2). Similarly, in patients undergoing PD, major complications (Clavien-Dindo ≥ 3a) were observed more frequently in LOPC (370/1206, 30,7%) than in MOPC (294/1123, 26,2%) and EOPC (14/83, 16,9%%; *p <* 0,01; Table 2). In DP, there was a trend of increased mortality and FTR rates from EOPC to MOPC to LOPC, yet not statistically significant. In PD, mortality significantly increased from EOPC (2,4%) to MOPC (3,6%) to LOPC (6,6%, *p <* 0,01). Additionally, significantly higher FTR rates could also be observed (EOPC 14,3%, MOPC 13,6%; LOPC 21,6%; *p <* 0,05).

### Risk predictors for major complications and FTR

To further analyze potential predictors regarding the risk of major complications (Clavien-Dindo ≥ 3a) logistic regression was calculated separately for PD and DP.

In the PD group, increasing age (OR 1,24 [95% CI 1,13—1,36]; *p* = 0,001) and BMI (OR 1,02 [95% CI 1,00—1,04]; *p* = 0,013) as well as an ASA score of 3 or 4 (OR 1,44 [95% CI 1,21—1,73]; *p* = 0,001) was associated with a significantly increased risk of major complications. An enlarged pancreatic duct > 3 mm (OR 0,75 [95% CI 0,60—0,92] *p* = 0,006) and hard pancreatic tissue (OR 0,69 [95% CI 0,56—0,84] *p* = 0,001) were associated with a significant reduction of major complications. Most important, hospitals certified as a pancreatic center by the DGAV, showed a significant reduction for the occurrence of major complications (Table 3).

**Table 3:** Risk of major complication in PD and DP

**Univariable logistic regression**			**Pancreaticoduodenectomy**			**Distal pancreatectomy**		
**Variable**	**Category/Units**		**OR**	**[95% CI]**	***P*-Value**	**OR**	**[95% CI]**	***P*-Value**
Age	Years/10		1.24	[1.13 - 1.36]	**0.001**	1.17	[0.97 - 1.40]	0.099
BMI	kg/m2		1.02	[1.00 - 1.04]	**0.013**	1.03	[0.99 - 1.08]	0.152
DGAV certification	No*	Yes	0.79	[0.65 - 0.94]	**0.010**	0.97	[0.64 - 1.46]	0.874
ASA	1/2*	3/4/5	1.44	[1.21 - 1.73]	**0.001**	1.09	[0.75 - 1.59]	0.650
Pancreatic duct	< 3mm*	> 3mm	0.75	[0.60 - 0.92]	**0.006**	1.07	[0.49 - 2.32]	0.872
Pancreatic texture	soft*	hard	0.69	[0.56 - 0.84]	**0.001**	0.62	[0.35 - 1.09]	0.095
Bilirubin	mg/dl		1.00	[0.99 - 1.02]	0.628	0.96	[0.89 - 1.04]	0.342
CA19-9	(U/ml)/100		1.00	[0.99 - 1.01]	0.186	1.00	[0.99 - 1.01]	0.999
CEA	n/ml		1.00	[1.00 - 1.01]	0.163	1.00	[1.00 - 1.00]	0.824
Diabetes mellitus	No*	Yes, no insulin	1.06	[0.81 - 1.37]	0.691	1.26	[0.75 - 2.14]	0.384
		Yes, insulin dependent	1.20	[0.94 - 1.53]	0.135	0.90	[0.49 - 1.66]	0.730
**Multivariable logistic regression**			**Pancreaticoduodenectomy**					
**Variable**	**Category/Units**		**OR**	**[95% CI]**	***P*-Value**			
Age	Years/10		1.18	[1.05 - 1.33]	**0.006**			
BMI	kg/m2		1.01	[1.00 - 1.03]	0.067			
DGAV certification	No*	Yes	0.85	[0.68 - 1.07]	0.175			
ASA	1/2*	3/4/5	1.39	[1.11 - 1.75]	**0.004**			
Pancreatic duct	< 3mm*	> 3mm	0.81	[0.64 - 1.02]	0.074			
Pancreatic texture	soft*	hard	0.75	[0.59 - 0.94]	**0.014**			

Predictors of major complications (Clavien–Dindo grade ≥ 3a). Results were derived from univariable and multivariable logistic regression including all patients in PD respectively DP group. Only predictors with *p <* 0,05 were included in the multivariable analysis

Asterix (*) indicates reference group, *CI* = confidence interval, *OR* = odds ratio

**Table 4:** Risk factors for FTR in PD and DP

**Univariable logistic regression**			**Pancreaticoduodenectomy**			**Distal pancreatectomy**		
Variable	Category/ Units		OR	[95% CI]	*P*-Value	OR	[95% CI]	*P*-Value
Age	Years/10		1.48	[1.17—1.87]	**0.001**	2.89	[1.16—7.25]	0.023
BMI	kg/m2		1.02	[0.99—1.04]	0.164	1.08	[0.93—1.25]	0.323
DGAV certification	No*	Yes	0.76	[0.51—1.13]	0.173	1.24	[0.31—4.93]	0.757
ASA	1/2*	3/4/5	2.30	[1.47—3.58]	**0.001**	1.15	[0.33—3.94]	0.829
Pancreatic duct	< 3mm*	> 3mm	1.02	[0.65—1.62]	0.929	0.42	[0.07—2.46]	0.336
Pancreatic texture	soft*	hard	0.74	[0.45—1.13]	0.151	0.48	[0.06—4.20]	0.510
Bilirubin	mg/dl		1.02	[0.98—1.05]	0.086	0.19	[0.01—2.84]	0.227
CA19-9	(U/ml)/100		1.01	[1.01—1.02]	**0.023**	0.98	[0.89—1.07]	0.639
CEA	n/ml		0.98	[0.98—1.01]	0.685	1.03	[0.99—1.06]	0.112
Diabetes mellitus	No*	Yes, no insulin	1.67	[0.98—2.84]	0.059	0.62	[0.07—5.26]	0.659
		Yes, insulin dependent	1.18	[0.70—1.199]	0.545	3.39	[0.77—14.90]	0.106
**Multivariable logistic regression**			**Pancreaticoduodenectomy**					
Variable	Category/ Units		OR	[95% CI]	*P*-Value			
Age	Years/10		1.40	[1.07—1.83]	**0.014**			
ASA	1/2*	3/4/5	2.11	[1.27—3.53]	**0**.**004**			
CA19-9	U/ml		1.00	[1.00—1.00]	**0.044**			

Predictors of FTR. Results were derived from univariable and multivariable logistic regression including all patients in PD respectively DP group with Clavien–Dindo grade ≥ 3a. Only predictors with *p <* 0,05 were included in the multivariable analysis

*CI* confidence interval, *OR* odds ratio

Asterix (*) indicates reference group

In multivariable regression analysis, age, ASA score and pancreatic texture were significantly associated with the risk of major complications.

In contrast to PD, no analyzed factor showed a significant association with the risk of major complications in DP (Table 3).

When analyzing predictors for FTR in the PD group, age (OR 1,48 [95% CI 1,16—1,87]; *p* = 0,001) and ASA score (OR 2,30 [95% CI 1,47 – 3,58]; *p* = 0,001) were associated with an increased risk of FTR. In the DP group, only age (OR 2,89 [95% CI 1,16 – 7,25]; *p* = 0,001) was associated with an increased risk of FTR (Table 4).

## Discussion

In the present study, we compared postoperative outcomes after pancreatic resections in patients with PDAC and explored differences between age groups by using the German nationwide pancreatic surgery registry (DGAV StuDoQ|Pancreas). After PD, older patients did not only suffer more frequently from complications in general, but also from major complications (Clavien-Dindo ≥ 3a). Comparing EOPC to LOPC, major complication rates almost doubled (16,9% vs. 30,7%) and mortality rates almost tripled (2,4% vs. 6,6%). The latter is mainly due to significantly higher failure-to-rescue rates in the elder population (21,6% vs. 14,3%). After DP we observed similar trends without reaching statistical significance. Furthermore, in both PD and DP, an increased age was associated with a higher burden of comorbidities and a prolonged stay in the hospital as well as on the ICU.

With regards to age, there is currently no unanimous definition of cut-off age values for the categories EOPC or LOPC. For EOPC, research groups have focused on patients younger than 45 [6] or even 40 years [19]. However, the majority included patients younger than 50 years of age [3, 5, 20] which lead us to use the same thresholds in this study for EOPC. Age greater than 70 years is usually defined as the cut off for the definition of elderly patients [21]; moreover, the median age of diagnosis for PDAC is 70 years [2]. Taken both facts together, we applied this reference age in our cohort and classified patients older than 70 years as LOPC.

When analyzing perioperative parameters, we surprisingly found a significantly lower median operation time from EOPC to LOPC in both resection groups. Although speculative, this might be related to more radical and thus, time-consuming, surgical approaches in patients with EOPC, due to more advanced and aggressive tumor stages, reflected by larger tumors and more advanced AJCC stages [6, 22, 23]. However, in this regard, tumor size and lymph node status did not differ significantly between the three age groups of our study population. Yet, this study lacks data on neoadjuvant chemotherapy, indicative of more advanced tumor stages or data on vessel or multivisceral resections, which also contribute to longer operational duration [24, 25].

In patients undergoing PD, CA19-9 values significantly increased from EOPC to LOPC. In the DP group, CA19-9 values increased with age, yet this change was not statistically significant. However, in PD, bilirubin values also increased with age. Since observational studies showed that cholestasis can cause elevated CA19-9 levels [26], it remains uncertain if the observed elevated CA19-9 values in the elder population are influenced by cholestasis or due to a different tumor biology. Additionally, in benign hepatobiliary conditions, age showed a significant correlation with elevated CA19-9 levels [27], indicating that differing CA19-9 levels might not be caused by different tumor biology amongst age groups.

Regarding pancreatic duct size, an enlarged pancreatic duct was observed more frequently in older age groups in the PD group. This may be part of a natural process as with increasing age, the pancreas can undergo an increase in density and firmness, accompanied by a uniform and widespread enlargement of the pancreatic duct [28]. Despite the known risk reduction of POPF associated with an enlarged pancreatic duct [29], typical complications after pancreatic resection such as POPF, DGE and PPH did not significantly differ between age or resection groups. Slightly higher rates of DGE were observed in the elderly, which is in line with findings of a meta-analysis that investigated outcomes after PD in octogenarians [30]. Higher rates of POPF after DP compared to PD are consistent with previous findings [31].

Our findings revealed both, an increasing age as well as an elevated ASA score as main predictors for the occurrence of major complications in PD. Elevated BMI, soft pancreatic tissue and a narrow pancreatic duct also increased the risk of major complications. These findings are in line with previous results which have demonstrated these risk factors to be major predictors of clinically relevant POPF [32]. In contrast, for patients who were treated in hospitals that were certified by the DGAV (German society of General and Visceral surgery) for pancreatic surgery, the occurrence of major complications was significantly reduced. It is well established that patients undergoing major pancreatic resections exhibit better outcomes when admitted to high volume hospitals [33] and a recent analysis showed that an increased number of pancreatic resections was associated with more frequent textbook outcomes in PD [34]. Consequently, certification was introduced to enhance said known positive effects of centralization in pancreatic surgery.

In contrast, and surprisingly, risk factors associated with major complications in PD did not predict major complications in DP. This might be due to a statistical effect caused by fewer case numbers in the DP group, smaller operational trauma and lack of pancreatic anastomosis in DP, leading to smaller differences between age groups or actually due to no effect.

It is known that after pancreatic resections, elder patients suffer more frequently from non-surgical complications such as pneumonia [35, 36] or cardiac events [30]. Also, higher FTR rates, defined as in-hospital mortality in patients with a major complication, lead to higher mortality [37]. In this context, our results demonstrate that with increasing age, FTR rates significantly increase in PD. In DP, a similar trend can be observed. Taken together, non-surgical complications occur more frequently in elderly patients, and when complications (surgical or non-surgical) occur, FTR is more likely. While our data show no association between DGAV certified centers and FTR, this might be influenced by heterogeneity, as certification only requires 40 pancreatic resections yet the cutoff for optimal outcomes just in PD might be even higher [38]. Furthermore, there is evidence that more specialized pancreatic centers with higher volumes are better prepared for the care of elderly and high-risk patients, demonstrating good outcomes despite complications [39].

Our findings support the concept that pancreatic resections in the elderly can be carried out safely. Importantly, pancreatic surgery-specific complications such as POPF, DGE or PPH do not occur more frequently in elder patients. However, higher FTR rates in LOPC lead to increased mortality. While our data indicate that elder patients can very well undergo pancreatic surgery with little surgery-specific complications, they should undergo treatment at specialized, high volume centers with top level expertise, because it reduces the likelihood major complications [38]. This is further highlighted by the fact that elder patients or patients treated at low-volume centers are less likely to undergo adjuvant chemotherapy [40, 41], yet it substantially improves overall survival [42]. Additionally, survival of elderly patients, when treated with chemotherapy is comparable to younger patients [43, 44].

Although a high number of patients was included, this study has limitations, in particular due to its retrospective nature. Furthermore, a vast majority of procedures was carried out as open surgeries, making it unclear if these results are transferable in an area of expanding minimally-invasive and robotic surgery [45]. Future research might address the question if especially elder patients benefit from minimally-invasive approaches and explore other factors that might reduce FTR rates in the elder.

## Conclusion

Age has a pronounced impact on the perioperative outcomes after pancreatic resections of PDAC. This effect is more prevalent in PD compared to DP. For the elder age group, the risk of major complications significantly increases, although surgery specific complications such as POPF, DGE or PPH do not occur more frequently. Furthermore, in this subgroup of older patients, mortality rates almost triple due to higher FTR rates. Apart from increasing age, critical risk factors for major postoperative complications after PD include an elevated ASA score and BMI as well as a soft pancreatic texture. Centralization of patients in high-volume centers could improve the outcome of pancreatic surgery, especially for older patients.

## Supplementary Information

Supplementary Material 1.

## Data Availability

Data extraction and analysis received approval from the StuDoQ steering committee and was conducted in compliance with StuDoQ data protection guidelines (StuDoQ-2019–0010). The Society for Technology, Methods, and Infrastructure for Networked Medical Research (TMF) approved the concept of informed consent and data safety. All data are property of the German Society of General and Visceral Surgery, but can be consulted at studoq@dgav.de upon request.

## Abbreviations

ASA: ASA physical status classification system
BMI: Body mass index
CA19-9: Tumor marker carbohydrate antigen 19–9
CEA: Tumor marker carcinoembryonic antigen
CI: Confidence interval
DGAV: German Society for General and Visceral Surgery (Deutsche Gesellschaft für Allgemein- und Viszeralchirurgie)
DGE: Delayed gastric emptying
DM: Diabetes mellitus
DP: Distal pancreatectomy
EOPC: Early-onset pancreatic cancer
FTR: Failure to rescue
ICD: International Classification of Diseases
ICU: Intensive care unit
ISGPS: International Study Group of Pancreatic Surgery
LOPC: Late-onset pancreatic cancer
MOPC: Middle-onset pancreatic cancer
OR: Odds ratio
PD: Pancreaticoduodenectomy
PDAC: Pancreatic ductal adenocarcinoma
POPF: Postoperative pancreatic fistula
PPH: Postpancreatectomy hemorrhage
PPPD: Pylorus-preserving pancreaticoduodenectomy
