# Supplementary material for: Perioperative outcomes in an age-adapted analysis of the German StuDoQ|Pancreas registry for PDAC
Source: BMC Surg. 2025 Jan 4;25:4. doi: 10.1186/s12893-024-02647-1 (PMC11700440; doi:10.1186/s12893-024-02647-1)
Supplement: Supplementary file 1 — Supplementary Material 1. [file 12893_2024_2647_MOESM1_ESM.docx]

**Table S1:** Extended table of perioperative variables and outcomes of patients with early-, middle- or late-onset pancreatic adenocarcinoma stratified regarding type of resection (PD vs. DP).

| **Type of surgery** | PD  (n = 2.412) | DP  (n = 599) | | Pancreaticoduodenectomy | | | Distal Pancreatectomy | | | *P* value |
| --- | --- | --- | --- | --- | --- | --- | --- | --- | --- | --- |
| **Onset**  n |  | |  | EOPC  83 | MOPC  1123 | LOPC  1.206 | EOPC  27 | MOPC  276 | LOPC  296 |  |
| **Median duration** (min) | 330  [272 - 403] | | 212  [163 - 269] | 352  [290 - 441] | 342  [278 - 415] | 320  [265 - 384] | 240  [165 - 315] | 218.5  [172 - 277] | 203  [155 - 253] | **P_PD_ < 0.01**  **P_DP_ < 0.01** |
| **Median stay**  **hospital**  (days) | 16  [12 - 23] | | 14.5  [11 - 22] | 15  [12 - 22] | 15  [12 - 21] | 17  [13 - 24] | 12  [11 - 18] | 14  [11 - 21] | 15  [12 -  23] | **P_PD_ < 0.01**  **P_DP_ < 0.05** |
| **Median stay ICU** (days) | 3  [1 - 5] | | 1  [1 - 3] | 2  [1 – 4] | 2  [1 – 4] | 3  [1 – 5] | 1  [0 – 2] | 1  [1 – 3] | 2  [1 – 4] | **P_PD_ < 0.01**  P_DP_ = 0.07 |
| **Resection status** |  | |  |  |  |  |  |  |  | P_PD_ = 0.87  P_DP_ = 0.69 |
| R0* | 918  (38.1%) | | 235  (39.2%) | 28 (33.7%) | 414  (36.9%) | 476  (39.6%) | 10  (37.0%) | 101  (36.6%) | 124  (41.9%) |  |
| R0 narrow  (< 1mm) | 335  (13.9%) | | 76  (12.7%) | 11 (13.3%) | 166  (14.8%) | 158  (13.1%) | 3  (11.1%) | 34  (12.3%) | 39  (13.2%) |  |
| R0 wide  (> 1mm) | 579  (24.0%) | | 137  (22.9%) | 23 (27.7%) | 275  (24.5%) | 281  (23.3%) | 9  (33.3%) | 71  (25.7%) | 57  (19.3%) |  |
| R1 | 526  (21.8%) | | 132  (22.0%) | 20 (24.1%) | 246  (21.9%) | 260  (21.6%) | 5  (18.5%) | 60  (21.7%) | 67  (22.6%) |  |
| R2 | 26  (1.1%) | | 5  (0.8%) | 1  (1.2%) | 11  (1.0%) | 14  (1.2%) | - | 2  (0.7%) | 3  (1.0%) |  |
| Unknown | 28  (1.2%) | | 14  (2.3%) | - | 11  (1.0%) | 17  (1.4%) | - | 8  (2.9%) | 6  (2.0%) |  |
| **POPF** |  | |  |  |  |  |  |  |  | P_PD_ = 0.48  P_DP_ = 0.35 |
| None | 2075  (86.0) | | 380  (63.4%) | 71  (85.5%) | 958  (85.3%) | 1046  (86.7%) | 18  (66.7%) | 174  (63.0%) | 188  (63.5%) |  |
| BL | 146  (6.1%) | | 78  (13.0%) | 7  (8.4%) | 75  (6.7%) | 64  (5.3%) | 6  (22.2%) | 31  (11.2%) | 41  (13.9%) |  |
| B | 106  (4.4%) | | 100  (16.7%) | 3  (3.6%) | 55  (4.9%) | 48  (4.0%) | 2  (7.4%) | 54  (19.6%) | 44  (14.9%) |  |
| C | 85  (3.5%) | | 41  (6.8%) | 2  (2.4%) | 35  (3.1%) | 48  (4.0%) | 1  (3.7%) | 17  (6.2%) | 23  (7.8%) |  |
| **CR-POPF** |  | |  |  |  |  |  |  |  | P_PD_ = 0.82  P_DP_ = 0.20 |
| No | 2221  (92.1%) | | 458  (76.5%) | 78  (94.0%) | 1033  (92.0%) | 1110  (92.0%) | 24  (88.9%) | 205  (74.3%) | 229  (77.4%) |  |
| Yes | 191  (7.9%) | | 141  (23.5%) | 5  (6.0%) | 90  (8.0%) | 96  (8.0%) | 3  (11.1%) | 71  (25.7%) | 67  (22.6%) |  |
| **PPH** |  | |  |  |  |  |  |  |  | P_PD_ = 0.57  P_DP_ = 0.77 |
| None | 2154  (89.3%) | | 570  (95.2%) | 76  (91.6%) | 1004  (89.4%) | 1074  (89.1%) | 26  (96.3%) | 261  (94.6%) | 283  (95.6%) |  |
| A | 49  (2.0%) | | 8  (1.3%) | 1  (1.2%) | 18  (1.6%) | 30  (2.5%) | - | 5  (1.8%) | 3  (1.0%) |  |
| B | 100  (4.1%) | | 10  (1.7%) | 3  (3.6%) | 43  (3.8%) | 54  (4.5%) | - | 6  (2.2%) | 4  (1.4%) |  |
| C | 109  (4.5%) | | 11  (1.8%) | 3  (3.6%) | 58  (5.2%) | 48  (4.0%) | 1  (3.7%) | 4  (1.4%) | 6  (2.0%) |  |
| **CR-PPH** |  | |  |  |  |  |  |  |  | P_PD_ = 0.82  P_DP_ =0.97 |
| No | 2203  (91.3%) | | 578  (96.5%) | 77  (92.8%) | 1022  (91.0%) | 1104  (91.5%) | 26  (96.3%) | 266  (96.4%) | 286  (96.6%) |  |
| Yes | 209  (8.7%) | | 21  (3.5%) | 6  (7.2%) | 101  (9.0%) | 102  (8.5%) | 1  (3.7%) | 10  (3.6%) | 10  (3.4%) |  |

| **DGE** |  |  |  |  |  |  |  |  | P_PD_ = 0.35  P_DP_ = 0.47 |
| --- | --- | --- | --- | --- | --- | --- | --- | --- | --- |
| None | 1929  (80.0%) | 529  (88.3%) | 73  (88.0%) | 902  (80.3%) | 954  (79.1%) | 24  (88.9%) | 247  (89.5%) | 258  (87.2%) |  |
| A | 243  (10.1%) | 43  (7.2%) | 7  (8.4%) | 109  (9.7%) | 127  (10.5%) | 3  (11.1%) | 21  (7.6%) | 19  (6.4%) |  |
| B | 160  (6.6%) | 16  (2.7%) | 3  (3.6%) | 78  (6.9%) | 79  (6.6%) | - | 5  (1.8%) | 11  (3.7%) |  |
| C | 80  (3.3%) | 11  (1.8%) |  | 34  (3.0%) | 46  (3.8%) | - | 3  (1.1%) | 8  (2.7%) |  |
| **CR-DGE** |  |  |  |  |  |  |  |  | P_PD_ = 0.14  P_DP_ = 0.06 |
| No | 2172  (90.0%) | 572  (95.5%) | 80  (96.4%) | 1011  (90.0%) | 1081  (89.6%) | 27  (100%) | 268  (97.1%) | 277  (93.6%) |  |
| Yes | 240  (10.0%) | 27  (4.5%) | 3  (3.6%) | 112  (10.0%) | 125  (10.4%) | - | 8  (2.9%) | 19  (6.4%) |  |
| **Clavien-Dindo** |  |  |  |  |  |  |  |  | **P_PD_ < 0.01**  P_DP_ = 0.24 |
| None | 1065  (44.2%) | 278  (46.4%) | 44  (53.0%) | 524  (46.7%) | 497  (41.2%) | 12  (44.4%) | 138  (50.0%) | 128  (43.2%) |  |
| Grade 1 | 227  (9.4%) | 61  (10.2%) | 8  (9.6%) | 117  (10.4%) | 102  (8.5%) | 6  (22.2%) | 25  (9.1%) | 30  (10.1%) |  |
| Grade 2 | 442  18.3%) | 120  (20.0%) | 17  (20.5%) | 188  (16.7%) | 237  (19.7%) | 6  (22.2%) | 52  (18.8%) | 62  (20.9%) |  |
| Grade 3a | 218  (9.0%) | 72  (12.0%) | 3  (3.6%) | 99  (8.8%) | 116  (9.6%) | - | 34  (12.3%) | 38  (12.8%) |  |
| Grade 3b | 231  (9.6%) | 34  (5.7%) | 5  (6.0%) | 112  (10.0%) | 114  (9.5%) | 2  (7.4%) | 16  (5.8%) | 16  (5.4%) |  |
| Grade 4a | 81  (3.4%) | 20  (3.3%) | 3  (3.6%) | 29  (2.6%) | 49  (4.1%) | 1  (3.7%) | 9  (3.3%) | 10  (3.4%) |  |
| Grade 4b | 26  (1.1%) | 3  (0.5%) | 1  (1.2%) | 14  (1.2%) | 11  (0.9%) | - | - | 3  (1.0%) |  |
| Grade 5 (death) | 122  (5.1%) | 11  (1.8%) | 2  (2.4%) | 40  (3.6%) | 80  (6.6%) | - | 2  (0.7%) | 9  (3.0%) |  |
| **Com-**  **plication** |  |  |  |  |  |  |  |  | **P_PD_ < 0.01**  P_DP_ = 0.18 |
| None/Minor  (Clavien-Dindo < 3a) | 1734  (71.9%) | 459  (76.6%) | 69  (83.1%) | 829  (73.8%) | 836  (69.3%) | 24  (88.9%) | 215  (77.9%) | 220  (74.3%) |  |
| Major (Clavien-Dindo ≥ 3a) | 678  (28.1%) | 140  (23.4%) | 14  (16.9%) | 294  (26.2%) | 370  (30.7%) | 3  (6.3%) | 61  (22.1%) | 76  (25.7%) |  |
| **Mortality** |  |  |  |  |  |  |  |  | **P_PD_ < 0.01**  P_DP_ = 0.13 |
| No | 2290  (94.9%) | 588  (98.2%) | 81  (97.6%) | 1083  (96.4%) | 1126  (93.4%) | 27  (100%) | 274  (99.3%) | 287  (97.0%) |  |
| Yes | 122  (5.1%) | 11  (1.8%) | 2  (2.4%) | 40  (3.6%) | 80  (6.6%) | - | 2  (0.7%) | 9  (3.0%) |  |
| **Failure to rescue** |  |  |  |  |  |  |  |  | **P_PD_ < 0.05**  P_DP_ = 0.21 |
|  | 122  (18.0%) | 11  (7.9%) | 2  (14.3%) | 40  (13.6%) | 80  (21.6%) | - | 2  (3.3%) | 9  (11.8%) |  |

Overall outcomes according to PD and DP and according to age group. Continuous data are shown as median, categorial data are shown as absolute (relative). Kruskal- Wallis test, Pearson’s chi square test and Fisher’s exact test used, comparing PD and DP resection within Early-, Middle- and Late-Onset group. Post hoc testing was calculated with Jonckheere-Terpstra test. *ICU* = intensive care unit, *POPF* = postoperative pancreatic fistula, *PPH* = postpancreatectomy hemorrhage, *DGE* = delayed gastric emptying; * = narrow or wide not indicated.

**Table S2:** Interaction between Age and ASA score.

| **Multivariable logistic regression** | | | **PD and DP** | | |
| --- | --- | --- | --- | --- | --- |
| **Variable** | **Category/**  **Units** |  | **OR** | **[95% CI]** | **P-Value** |
| Age | Years/10 |  | 1.28 | [1.13 - 1.45] | **< 0.001** |
| ASA | 1/2* | 3/4 | 3.13 | [0.97 - 10.13] | 0.057 |
| Age * ASA |  |  | 0.14 | [0.75 - 1.04] | 0.136 |

Interaction analysis for Age and ASA score. Asterix (*) indicates reference group, *CI* = confidence interval, *OR* = odds ratio.
